# Analysis of the Function and Regulatory Mechanisms of the *AmFLS* Gene, Encoding the Flavonol Synthase in *Abelmoschus manihot* L.

**DOI:** 10.3390/plants15152321

**Published:** 2026-07-28

**Authors:** Dayun Wang, Hongtao Chu, Zhongxu Liu, Yidong Song, Xiangyi Wang, Yuying Li, Junli Wang, Lai Wei, Shengtai Shi, Shishuo Zhang, Li Cao

**Affiliations:** 1 Agricultural College, Yanbian University, Yanji 133002, China; dayunwang6@163.com (D.W.); chuhongtao2001@163.com (H.C.); 19997199021@163.com (Z.L.); 2024050906@ybu.edu.cn (X.W.); 2024050899@ybu.edu.cn (Y.L.); 2025050937@ybu.edu.cn (J.W.); 15844978189@163.com (L.W.); 13331753453@163.com (S.S.); a246800907@163.com (S.Z.); 2 Yanbian Prefecture Agricultural Biological Resources and Rural Energy Management Station, Yanji 133000, China; yidong1023@outlook.com

**Keywords:** *Abelmoschus manihot* L., flavonol, *AmFLS*, *AmMYB111*, AmRGL2

## Abstract

*Abelmoschus manihot* L. (Jinhuakui, JHK) is a Malvaceae plant with ornamental, medicinal, and edible value. It is rich in flavonoids, with a particularly high content of flavonols; however, current research on the molecular mechanisms underlying flavonol biosynthesis in JHK, particularly the upstream transcriptional regulatory networks, remains limited. In this study, JHK was used as the model plant to determine the patterns of flavonol accumulation in different tissues and during the flowering period. We cloned the *AmFLS* gene to validate its function and screened for and identified its upstream transcriptional regulators and interacting proteins. The results indicate that flavonols in JHK are primarily concentrated in floral organs, with the highest level observed during the budding stage. Furthermore, we found that *AmFLS* positively regulates flavonol biosynthesis and serves as the central rate-limiting gene. Additionally, *AmMYB111* is a nuclear-localized R2R3-MYB repressor that directly binds to the *AmFLS* promoter and inhibits its transcription, thereby negatively regulating flavonol accumulation. Finally, the DELLA family proteins AmRGL2 and AmMYB111 can specifically interact with each other and actively promote the accumulation of flavonols. This study provides a preliminary elucidation of the molecular mechanism by which upstream transcriptional regulation of the *AmFLS* gene controls flavonol biosynthesis, offering a theoretical basis for further elucidating the regulatory network of flavonol metabolism in JHK and accelerating its industrial development.

## 1. Introduction

*Abelmoschus manihot* L. is also known as “caifurong” (CFR) and “jinronghua” (JRH) in Chinese. It can grow up to 2 m tall and has a well-developed root system. Its stems are sturdy and woody at the base, and its leaves are alternate. The flowers are as large as bowls, with golden-yellow corollas bearing purple spots at the base [1,2]. JHK is rich in bioactive compounds, and every part of the plant can be used medicinally. Its main components include flavonoids, unsaturated fatty acids, polysaccharides, trace elements, and alkaloids. It possesses antioxidant, lipid-lowering, anti-inflammatory, antipyretic, analgesic, and immune-boosting properties [3,4,5,6].

In plants, flavonols play a key role in stress responses and growth and development. Under drought stress, the flavonol biosynthesis pathway in peanut guard cells is specifically activated by severe drought, promoting the accumulation of quercetin and kaempferol [7,8,9]. These flavonoids not only directly scavenge excess reactive oxygen species but also upregulate the expression of *AhNCED1*, a key gene in ABA synthesis. By activating the stomatal closure signaling pathway, they enhance the water use efficiency of peanut plants, thereby significantly improving their drought tolerance [10]. Due to their potent antioxidant and anti-inflammatory properties, flavonols have become a focal point of research in the fields of nutritional health and drug development. Quercetin and kaempferol can improve lipid metabolism, regulate blood pressure and blood glucose levels, and reduce the risk of cardiovascular disease. They also play a role in preventing atherosclerosis and thrombosis and promoting vasodilation [11,12,13,14].

Flavonol synthase (*FLS*) is the central rate-limiting enzyme in the flavonoid metabolic pathway and belongs to the Fe^2+^/2-oxoglutarate-dependent dioxygenase (2-ODD) family. The dihydroflavonol dehydrogenation reaction it catalyzes is a key step in flavonol biosynthesis. Additionally, by competing with dihydroflavonol reductase (DFR) for the common substrate dihydroflavonol, it acts as a “metabolic switch” regulating the synthesis of flavonols, anthocyanins, and proanthocyanidins, directly determining the composition and proportions of plant secondary metabolites [15,16,17]. The conserved structure of *FLS* genes confers diverse physiological functions across different plant species, with their core roles centered on two major aspects: color formation and stress resistance. By regulating the balance between the synthesis of flavonols and anthocyanins, *FLS* genes contribute to the fine-tuning of plant flower color [18,19]. In Magnolia *denudata*, the expression peak of the *YdFLS* gene coincides with the accumulation of flavonols in the flowers. In contrast, in Magnolia *liliiflora*, the expression of *YlFLS* gradually declines during flower development, leading to an increase in the relative content of anthocyanins. These expression patterns ultimately result in the distinct floral color differences between the two species. In terms of stress resistance, the function of *FLS* is primarily mediated by its corresponding stress-responsive metabolite, flavonol [20,21,22]. In Arabidopsis, the *AtFLS1*, *AtFLS3*, and *AtFLS4* triple mutant, due to severely impaired flavonol synthesis, exhibits UV-B radiation, with its photosynthetic efficiency decreasing by 42% compared to the wild type under UV-B treatment. In contrast, plants overexpressing the *AtFLS* genes exhibit enhanced UV-B tolerance [23]. This is attributed to the fact that flavonoid components in the leaves, such as quercetin and kaempferol, effectively absorb UV-B radiation and scavenge radiation-induced reactive oxygen species, thereby reducing DNA damage and disruption of the photosynthetic system [24,25,26,27].

DELLA proteins are a class of nuclear proteins widely distributed in plants that are regulated by GA signaling; they also serve as central negative regulators in the GA signaling pathway and play a key role in physiological processes such as plant growth and development, secondary metabolite synthesis, and stress responses [28]. In Arabidopsis, DELLA proteins interact with MYB21 and MYB24 and regulate filament elongation by attenuating their transcriptional activity [29]. In addition, DELLA proteins (RGA and GAI) interact with SG7 MYB proteins (MYB12 and MYB111) both in vitro and in vivo, thereby enhancing the binding affinity of MYB proteins to the promoter regions of key genes involved in flavonoid biosynthesis, which in turn increases the transcriptional levels of these genes [30]. DELLA proteins directly sequester MYBL2 and JAZ repressors, leading to the release of bHLH/MYB subunits and subsequently to the formation of an active MBW complex, which then activates the anthocyanin biosynthetic pathway [31].

To fill the research gap regarding the upstream transcriptional regulatory network governing the biosynthesis of flavonols in JHK, we used JHK as a model organism to determine the spatiotemporal distribution patterns of flavonols in different organs and across different stages of the flowering period. We cloned and identified AmFLS, a key gene in the flavonol biosynthesis pathway, and conducted a systematic study focusing on its gene function, promoter regulatory elements, the upstream transcription factor AmMYB111, and the interacting protein AmRGL2. This study provides a theoretical foundation for refining the molecular regulatory network of flavonoid biosynthesis in JHK.

## 2. Results

### 2.1. Analysis of Variations in Flavonol Content in Different Tissues and During Different Flowering Stages of JHK

**Figure 1:**
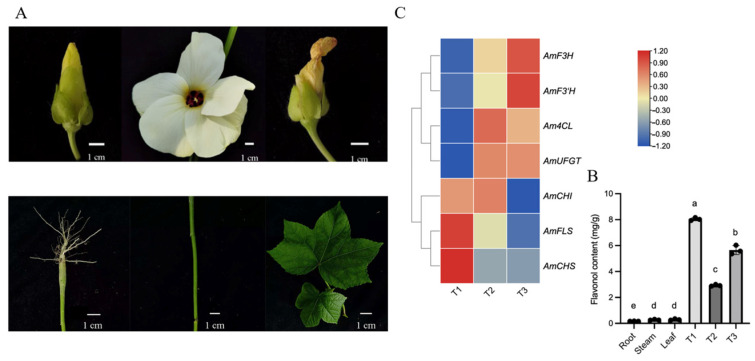
Analysis of flavonol content and expression of key genes in the flavonol biosynthetic pathway in different tissues of JHK during the flowering period. (**A**) Phenotypic diagrams of different flowering stages and plant parts of JHK (Bar = 1 cm); (**B**) Flavonol content in different tissues (R: Root; St: Stem; L: Leaf; T1: Bud stage; T2: Full flowering stage; T3: Withering stage); (**C**) Heat map of relative expression levels of 7 key genes in the flavonol synthesis pathway at different flowering stages. Different lowercase letters indicate significant differences (*p* < 0.05).

To elucidate the tissue-specific and developmental-stage-specific patterns of flavonol biosynthesis in JHK, this study selected floral organs at different stages of flowering (bud stage T1, full-flowering stage T2, and withering stage T3) as well as vegetative organs, including stems (St), leaves (L), and roots (R) (Figure 1A). The flavonol content in these tissues was measured. The results showed that flavonols exhibited distinct distribution patterns across the various tissues of JHK. Flavonols were significantly enriched in floral organs, with the highest concentration of 8.04 mg/g observed during the bud stage (T1). The concentration followed a trend of initially decreasing and then increasing as flowering progressed, reaching 2.93 mg/g at full bloom (T2) and 5.66 mg/g during the petal-fall stage (T3). Flavonol content in vegetative organs was significantly lower than in floral organs, ranging from 0.18 to 0.31 mg/g; specifically, flavonol levels in stems (St) and leaves (L) were similar, while roots (R) had the lowest content (Figure 1B).

These significant fluctuations in flavonol content in flowers indicate that flavonol biosynthesis is subject to strict temporal regulation during the flowering period. To elucidate the regulatory mechanisms, this study conducted quantitative expression analysis of seven key genes in the flavonol biosynthesis pathway. The results showed that the expression levels of the *AmFLS* and *AmF3′H* genes differed significantly across the three flowering stages, whereas the expression levels of upstream or side-branch pathway genes such as *AmCHI*, *Am4CL*, *AmCHS*, and *AmUFGT* did not show significant differences between T1 and T2 or between T2 and T3 (Figure 1C). From the perspective of metabolic pathways, *AmF3′H* and *AmFLS* are direct branching point enzymes in flavonol biosynthesis, suggesting that these two may be the core key genes for flavonol biosynthesis in JHK.

### 2.2. Cloning of the AmFLS and AmF3′H Genes in JHK

The CDS sequences of the *AmFLS* and *AmF3′H* genes were obtained from transcriptomic data. The coding regions of the *AmFLS* and *AmF3′H* genes were 1010 bp and 986 bp in length (Figure S1), respectively. Specific primers were designed according to standard primer design principles. Using cDNA synthesized by reverse transcription of total RNA extracted from JHK leaves as a template, fragment amplification was performed via PCR. Analysis by agarose gel electrophoresis revealed a bright band at approximately 1000 bp in the PCR product, which is consistent with the size of the target gene. The PCR product was purified, ligated into the pCE2-TA/Blunt-Zero vector, and transformed into *Escherichia coli* DH5α-competent cells. Positive clones were selected for sequencing and identification; the sequencing results matched the target gene sequence, confirming successful gene cloning.

### 2.3. Functional Validation and Expression Characteristics of the FLS and F3′H Genes

#### 2.3.1. Construction of Vectors for Virus-Induced Silencing of the AmFLS and AmF3′H

Using the *AmFLS* and *AmF3′H* plasmids, which had been sequenced as correct, as templates, we designed homologous-arm primers and amplified the gene-silencing fragment via PCR. After ligation into the vector, the construct was transformed into *Escherichia coli* DH5α-competent cells, and positive clones of approximately 300 bp in size were selected (Figure S2). Following confirmation of their correctness by sequencing, they were transformed into *Agrobacterium* EHA105.

#### 2.3.2. Construction of AmFLS and AmF3′H Transient Overexpression Vectors

Using the same method, the full-length CDS sequences of *AmFLS* and *AmF3′H* were cloned into the pCAMBIA2300 vector, transformed into *Escherichia coli* DH5α-competent cells, and positive clones of approximately 2000 bp in size were selected (Figure S3). After confirmation by sequencing, these were transformed into *Agrobacterium*.

#### 2.3.3. Analysis of the Expression Patterns of the AmFLS and AmF3′H Genes in Different Tissues and Flowering Stages, and Their Effects on Flavonol Content

**Figure 2:**
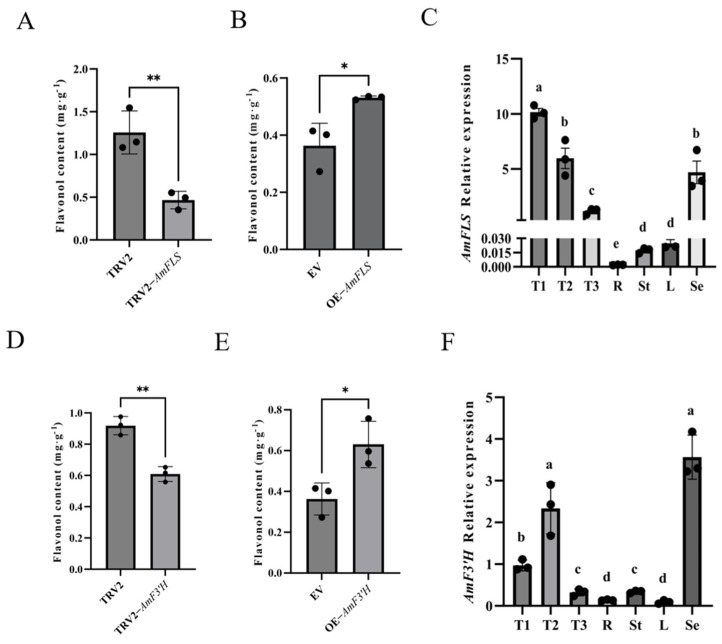
Gene function validation and expression pattern analysis: (**A**) flavonol content in the TRV2-*AmFLS* silenced line; (**B**) flavonol content in OE-*AmFLS* overexpression line; (**C**) relative expression level of the *AmFLS* gene in different tissues and flowering stages; (**D**) flavonol content in the TRV2-*AmF3′H* silenced line; (**E**) flavonol content in OE-*AmF3′H* overexpression line; (**F**) relative expression level of *AmF3′H* gene in different tissues and flowering stages (R: Root; St: Stem; L: Leaf; Se: Seed; T1: Bud stage; T2: Full flowering stage; T3: Withering stage). Different lowercase letters indicate significant differences (*p* < 0.05), * indicates significant differences (*p* < 0.05), ** indicates extremely significant differences (*p* < 0.01).

To investigate the effects of *AmFLS* and *AmF3′H* on flavonol biosynthesis in JHK, silencing and overexpression experiments were conducted using a gene functional validation system in JHK leaves. In both experiments, there were no significant differences in plant phenotypes (Figure S4). Analysis of flavonol content in the leaves revealed that flavonol levels in the silenced groups of all three genes were significantly lower than those in the TRV2 control group (Figure 2A,D), while flavonol levels in the overexpression groups were significantly higher than those in the EV control group (Figure 2B,E). These results indicate that all three genes promote flavonol synthesis in JHK.

To analyze the spatiotemporal expression patterns of *AmFLS* and *AmF3′H*, qRT-PCR was performed on the aforementioned tissue samples. The results showed that the *AmF3′H* gene exhibited the highest expression level in seeds (Se), while expression levels were relatively low in other vegetative organs, such as roots (R), stems (St), and leaves (L). In floral organs, expression levels were high during the bud stage (T1) and full bloom (T2) and lowest during the petal fall stage (T3) (Figure 2F). These results suggest that *AmF3′H* is not only a core gene involved in flavonol biosynthesis in floral organs but also plays a key role in flavonol metabolism in storage organs such as seeds. *AmFLS* is highly expressed in flowers and seeds (Se), whereas its expression levels are extremely low in vegetative organs such as roots (R), stems (St), and leaves (L) (Figure 2C). Furthermore, in floral organs, *AmFLS* expression gradually decreases with floral development, declining from high expression during the bud stage (T1) to the lowest expression during the petal-shedding stage (T3). This dynamic pattern suggests that this gene may play a key role in flavonol accumulation during early floral development. Combined with its high expression in seeds, it is speculated that *AmFLS* is not only a core component in flavonol accumulation in floral organs but may also be involved in the storage and metabolism of flavonols during seed development. Furthermore, *AmFLS* is a key rate-limiting enzyme in the flavonol biosynthetic pathway; compared to *AmF3′H*, silencing this gene resulted in an approximately 62.94% decrease in flavonol content, therefore warranting further investigation into its underlying regulatory mechanisms. In summary, *AmFLS* functions as the core rate-limiting gene in flavonol biosynthesis and shows a more significant regulatory effect. Therefore, the subsequent study focused on the transcriptional regulatory mechanism of *AmFLS*, while *AmF3′H* will be further explored in future research.

Furthermore, as flowering progresses, AmFLS expression levels continue to decline, reaching a minimum at the T3 stage, while the flavonoid content begins to rise at the same time (Figure 1B and Figure 2C). This apparent inconsistency between gene expression levels and flavonoid content is not contradictory. On the contrary, it suggests that flavonoid accumulation during the T3 stage is not due to *AmFLS*-mediated de novo biosynthesis. Instead, it is more likely associated with the transport and redistribution of flavonoids during floral organ senescence, enhanced metabolic stability, or the compensatory activation of alternative flavonoid biosynthetic pathways. This further suggests that *AmFLS* primarily acts as a core rate-limiting factor during the T1 and T2 stages and is a key gene in early flavonoid biosynthesis. qRT-PCR analysis confirmed that the transcript levels of *AmFLS* and *AmF3′H* were significantly reduced in the TRV2-silenced lines and significantly elevated in the OE-overexpression lines, compared to their respective controls (Supplementary Figure S4).

### 2.4. Screening and Cloning of Upstream Regulatory Transcription Factors in AmFLS

#### 2.4.1. AmFLS Promoter Cloning and Sequence Validation

To elucidate the transcriptional regulatory mechanism of the *AmFLS* gene, this study first cloned a promoter sequence (pro*AmFLS*) approximately 500 bp upstream of the gene from the genomic DNA of JHK using the chromosome walking method. The purified PCR products were verified by sequencing. The results confirmed that this sequence seamlessly connects to the upstream region of the *AmFLS* coding region, indicating that it is the correct target promoter sequence (Figure S5A,C).

#### 2.4.2. Prediction and Analysis of Cis-Acting Elements

Analysis of the pro*AmFLS* sequence using the Plant CARE online tool (https://bioinformatics.psb.ugent.be/webtools/plantcare/html/, accessed on 15 August 2025.) (Figure S5B) revealed that, in addition to core promoter elements (e.g., TATA-box and CAAT-box), the sequence is enriched with various regulatory elements. These include *MYB* and Myb-binding sites, which provide binding sites for direct *MYB* transcription factor interaction. The promoter also contains stress and hormone response elements, such as the anaerobic response element (ARE) and the GA response element (TATC-box), as well as light-response-related motifs, including the 3-AF1 binding site and the TCT-motif. Additionally, other regulatory elements like MYC and the AAGAA-motif were identified.

The presence of MYB-binding sites suggests that the expression of the *AmFLS* gene may be directly regulated by *MYB* family transcription factors. Given that *MYB* is also extensively involved in plant secondary metabolism, this provides a clear direction for the subsequent screening of its upstream regulatory factors. Furthermore, the enrichment of various regulatory elements indicates that *AmFLS* expression is subject to multiple regulatory pathways, including hormonal signaling, reflecting the complexity of the regulatory network governing flavonol biosynthesis in JHK.

#### 2.4.3. Screening and Cloning of Upstream MYB Transcription Factors

**Figure 3:**
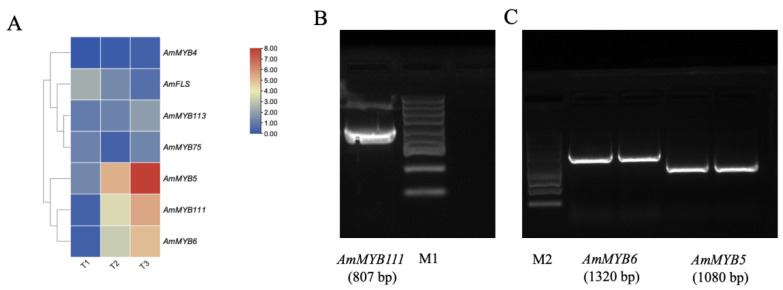
Screening and cloning of *MYB* transcription factors upstream of *AmFLS* gene: (**A**) heatmap of expression correlation between the *MYB* family genes and *AmFLS* gene; (**B**) electrophoresis of *AmMYB111* gene cloning; (**C**) electrophoresis of *AmMYB6* and *AmMYB5* gene cloning. M1 and M2 are the DNA molecular weight markers, Marker = 5000 bp.

Based on the MYB cis-regulatory elements in pro*AmFLS*, combined with the JHK transcriptome database and the temporal expression patterns of the *AmFLS* gene in flowers (Figure 3A), this study identified three candidate MYB transcription factors, *AmMYB111*, *AmMYB6*, and *AmMYB5*, which show a high correlation with the *AmFLS* expression pattern. Amplification was performed using PCR, and successful sequencing alignment confirmed that the three *MYB* transcription factors obtained were 807 bp, 1320 bp, and 1080 bp in size, respectively (Figure 3B,C).

### 2.5. AmMYB111 Directly Binds to the AmFLS Promoter

#### 2.5.1. Yeast One-Hybrid (Y1H) Assay

**Figure 4:**
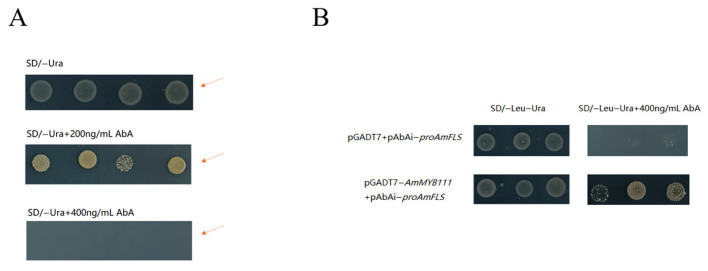
Yeast one-hybrid (Y1H) assay: (**A**) screening for minimum inhibitory concentration of AbA against Y1H Gold strain; (**B**) validation of the specific binding between *AmMYB111* and *AmFLS* promoter by yeast one-hybrid assay.

In a yeast one-hybrid assay, pro*AmFLS* was cloned into the pAbAi reporter vector, linearized, and transformed into the Y1HGold strain. Gradient screening determined that its minimum inhibitory concentration (MIC) for AbA was 400 ng·mL^−1^ (Figure 4A). Subsequently, the *MYB* transcription factor coding region was cloned into the pGADT7 effector vector and co-transformed into yeast with the pAbAi-pro*AmFLS* reporter vector. The results showed that on SD/-Leu-Ura screening medium supplemented with 400 ng·mL^−1^ AbA, among the three experimental groups, only the co-transformed pGADT7-*AmMYB111* + pAbAi-pro*AmFLS* strain grew normally, whereas the control group with the empty vector pGADT7 + pAbAi-pro*AmFLS* failed to grow (Figure 4B), indicating that *AmMYB111* is capable of specifically binding to the *AmFLS* promoter in yeast.

#### 2.5.2. Dual-Luciferase Reporter System (Dual-LUC)

**Figure 5:**
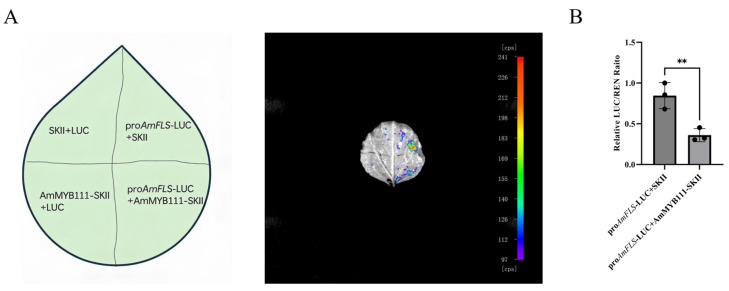
In vivo imaging and quantitative analysis of dual-luciferase reporter assay (Dual-Luc): (**A**) dual-luciferase reporter in vivo imaging; (**B**) quantitative analysis. ** indicates an extremely significant difference (*p* < 0.01).

To further validate its regulatory function in plants, the 0800-LUC-pro*AmFLS* reporter vector and the pGreenII 62-SK-*AmMYB111* effector vector were constructed and co-transformed into *Nicotiana benthamiana* leaves using *Agrobacterium*-mediated transient transformation. Live-cell imaging results showed that the fluorescence signal in tobacco leaves co-transformed with pGreenII 62-SK-*AmMYB111* and 0800-LUC- pro*AmFLS* was significantly weaker than that in the empty vector control group (Figure 5A). Quantitative analysis indicated that the LUC/REN ratio in the experimental group was significantly lower than that in the control group (Figure 5B). These results confirm that *AmMYB111* not only directly binds to the *AmFLS* promoter but also significantly inhibits its transcriptional activity in plants, acting as an upstream negative regulator of *AmFLS*.

The results of the two experiments described above indicate that *AmMYB111* participates in the molecular mechanism regulating flavonol biosynthesis in JHK. It does so by directly targeting and binding to the *AmFLS* promoter, thereby inhibiting its transcription. This finding provides an important functional node for elucidating the transcriptional regulatory network of luteolin biosynthesis in JHK.

### 2.6. Analysis of the Expression Pattern and Functional Validation of AmMYB111 Transcription Factor

#### 2.6.1. Analysis of the Subcellular Localization of AmMYB111

**Figure 6:**
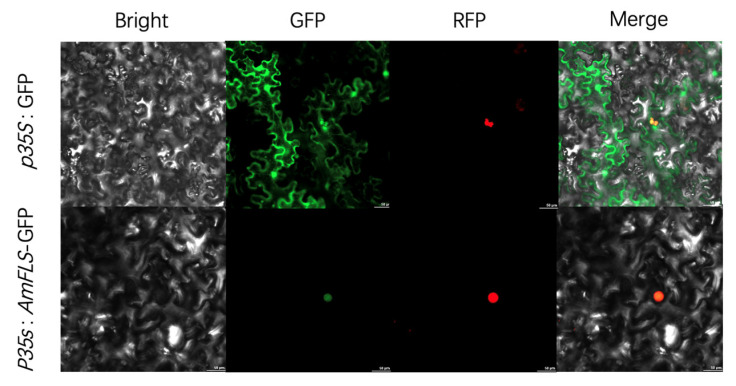
Subcellular localization analysis of AmMYB111 protein. p35S: GFP represents the empty vector control group; p35S: *AmMYB111*-GFP represents the AmMYB111-GFP fusion protein; Bright: bright field; GFP: green fluorescent protein; RFP: red fluorescent protein (nuclear localization marker); Merge: merged image of bright field and fluorescence channels; Bar = 50 μm.

To determine the subcellular localization of the AmMYB111 protein, this study introduced the 35S: *AmMYB111*-GFP recombinant vector, labeled with a nuclear marker, into *Nicotiana benthamiana* leaves via *Agrobacterium*-mediated transformation, using the 35S: GFP empty vector labeled with a nuclear marker as a control. Confocal microscopy revealed that the GFP signal in the control group was widely distributed throughout the cell, whereas the green fluorescence of *AmMYB111*-GFP completely overlapped with the nuclear marker signal and was specifically localized to the cell nucleus (Figure 6). These results indicate that AmMYB111 is a typical nuclear-localized protein, consistent with the characteristic of transcription factors exerting regulatory functions within the cell nucleus.

#### 2.6.2. Analysis of the Expression Patterns of AmMYB111 in Different Tissues and During Different Stages of Flowering

**Figure 7:**
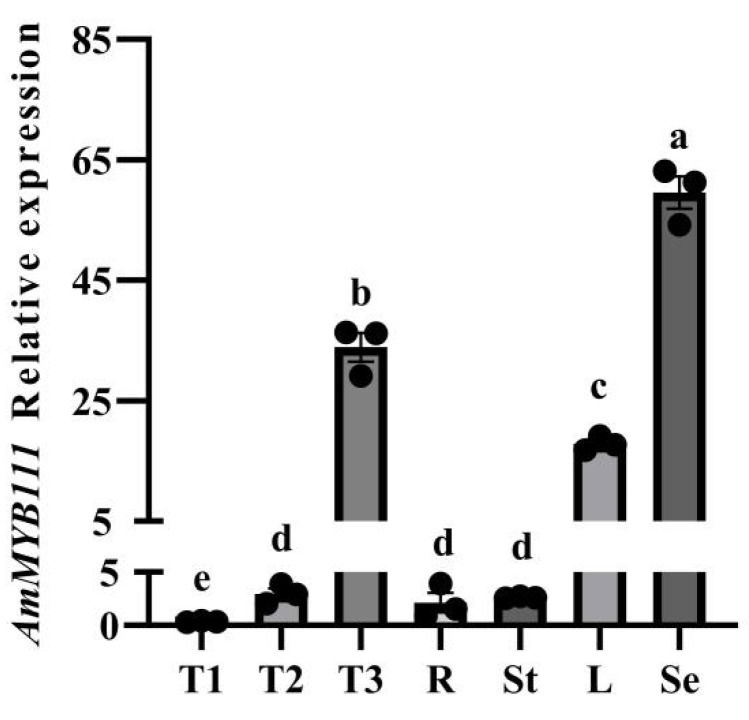
Expression analysis of *AmMYB111* gene in different tissues and flowers at different flowering stages: T1: Bud stage; T2: Full flowering stage; T3: Withering stage; R: Root; St: Stem; L: Leaf; Se: Seed. Different lowercase letters indicate significant differences (*p* < 0.05).

To investigate the spatiotemporal expression patterns of *AmMYB111* in JHK, this study used qRT-PCR to detect *AmMYB111* expression levels in different phytonutrient tissues and floral organs at various developmental stages. The results showed that *AmMYB111* was expressed in all tested tissues, with the highest expression level in seeds (Se), followed by leaves (L) and stems (St), while the expression level in roots (R) was extremely low. In floral organs, its expression level gradually increased with development, rising from low levels at the early flowering stage (T1) to a peak at the petal abscission stage (T3) (Figure 7). It is worth noting that the expression pattern of *AmMYB111* across different stages of flowering shows a significant negative correlation with the gradually decreasing expression pattern of *AmFLS* in the flower. This negatively correlated expression pattern is fully consistent with the previously confirmed finding that *AmMYB111* acts as a repressor of *AmFLS* transcription; this experiment further validates the role of *AmMYB111* as an upstream negative regulator of *AmFLS* in the flavonol biosynthesis process in JHK.

#### 2.6.3. Screening for Proteins That Interact with AmMYB111

**Figure 8:**
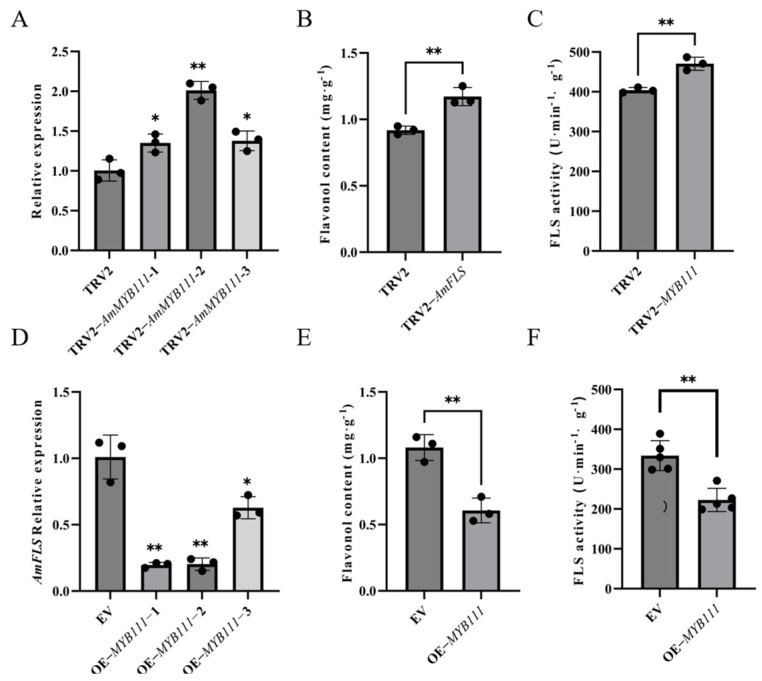
Functional verification of *AmMYB111* transcription factor: (**A**) Relative expression level of *AmFLS* gene in TRV2−*AmMYB111* silenced lines. (**B**) *FLS* enzyme activity in TRV2−*AmMYB111* silenced lines. (**C**) Flavonol content in TRV2−*AmMYB111* silenced lines. (**D**) Relative expression level of *AmFLS* gene in OE−*AmMYB111* overexpressed lines. (**E**) Flavonol content in OE−*AmMYB111* overexpressed lines. (**F**) *FLS* enzyme activity in OE−*AmMYB111* overexpressed lines. * Indicates significant difference (*p* < 0.05), ** indicates extremely significant difference (*p* < 0.01).

To validate the regulatory function of *AmMYB111* in vivo, this study constructed the *AmMYB111* transient overexpression vector pCAMBIA2300-*AmMYB111* and the VIGS silencing vector TRV2-*AmMYB111*, which were then introduced into JHK leaves via *Agrobacterium*-mediated transient transformation. In both experiments, there were no significant differences in plant phenotypes (Figure S6). qRT-PCR verification confirmed that *AmMYB111* was successfully silenced in the TRV2-*AmMYB111* lines and overexpressed in the OE-*AmMYB111* lines (Supplementary Figure S6A,C). In the silencing experiment, qRT-PCR results showed that, compared with the TRV2 control group, the TRV2-*AmMYB111* transformation group was significantly reduced, while the expression of its downstream target gene *AmFLS* was significantly increased (Figure 8A). Analysis of flavonol content in the leaves of the silenced lines revealed that flavonol levels were significantly higher in the silenced lines than in the TRV2 control group (Figure 8B). In the overexpression experiments, the OE-*AmMYB111* transformation group showed results opposite to those in the silencing experiments when compared to the EV control group. Specifically, the expression level of *AmMYB111* was significantly increased, while that of its downstream target gene *AmFLS* was significantly decreased. The flavonol content in the leaves of the overexpression lines was also significantly lower compared to the EV control group (Figure 8D,E). These two experiments clearly confirm that *AmMYB111* negatively regulates flavonol biosynthesis in JHK.

To gain a deeper understanding of the molecular mechanisms by which *AmMYB111* regulates flavonol biosynthesis, this study further measured the activity of *FLS*, a key enzyme in flavonol biosynthesis, in JHK leaves. The results showed that *FLS* activity was higher in the TRV2-*AmMYB111* silencing group than in the TRV2 control group (Figure 8C). In the OE-*AmMYB111* overexpression group, *FLS* activity was decreased compared to the EV control group (Figure 8F). This indicates that *AmMYB111* not only downregulates *AmFLS* expression by directly binding to its promoter but also indirectly reduces the activity of FLS. FLS is the key enzyme in flavonol biosynthesis in JHK. Consequently, *AmMYB111* inhibits flavonol biosynthesis at the enzymatic level.

### 2.7. Screening and Validation of AmMYB111 Interacting Proteins

#### 2.7.1. The Effect of AmMYB111 on AmFLS Expression and Flavonol Content

In the transcriptional regulatory pathways governing the synthesis of plant flavonols, R2R3-MYB transcription factors exhibit functional diversity. Flavonol-specific *MYB*s typically activate the expression of downstream structural genes independently, whereas flavonol branches such as anthocyanins and proanthocyanidins rely more heavily on the MYB-bHLH-WD40 (MBW) ternary complex to achieve precise spatiotemporal regulation. To comprehensively elucidate the regulatory mechanism of *AmMYB111* in JHK, we first utilized phylogenetic tree analysis to predict interactions of its homolog, Raymondia *MYB6*, via the STRING database (https://string-db.org/). This analysis also aimed to determine whether *AmMYB111* functions by forming protein complexes. The results indicate potential interactions with HTH-MYB domain proteins, MYND-domain proteins, and others (Figure S7A,B). Second, we preselected AmTTG1 and AmRGL2 as the two candidate proteins most likely to interact with AmMYB111. This selection was based on (1) existing research on MYB-mediated flavonol biosynthesis in model plants such as Arabidopsis and cotton, (2) the transcriptomic data of JHK, and (3) the fact that regulatory factors in the flavonol pathway and their downstream structural genes typically exhibit synergistic transcriptional regulation patterns. We then proceeded to clone and validate the sequences of these candidate genes.

Specific amplification primers were designed based on the CDS sequences obtained from the JHK transcriptome. Using cDNA from petals at peak flowering as a template, the full-length coding regions of the two candidate genes were amplified via PCR. Sequencing results showed that the full-length CDS of *AmTTG1* is 1047 bp, encoding 348 amino acids, and belongs to the WD40 family; the full-length CDS of *AmRGL2* is 558 bp, encoding 185 amino acids, and belongs to the DELLA family (Figure S7C). Both genes possess typical conserved domains associated with the regulation of plant flavonol metabolism.

#### 2.7.2. Yeast Two-Hybrid (Y2H)

**Figure 9:**
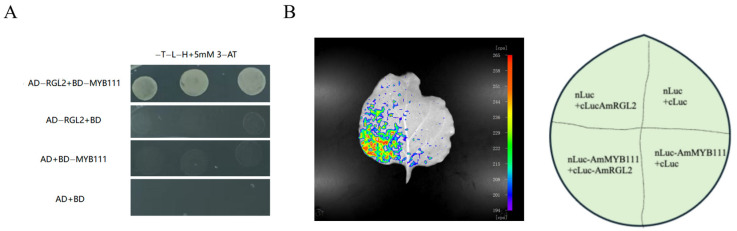
Verification of protein interaction between AmMYB111 and AmRGL2: (**A**) verification of the interaction between AmMYB111 and AmRGL2 by yeast two-hybrid (Y2H) assay; (**B**) verification of the interaction between AmMYB111 and AmRGL2 in *Nicotiana Benthamiana* leaves by luciferase complementation assay (LCA).

Yeast two-hybrid (Y2H) and luciferase complementation assay (LCA) were used to validate the interaction between the candidate proteins and AmMYB111. The Y2H assay results showed that the experimental yeast strain containing AmMYB111 and AmRGL2 grew normally on a screening medium lacking Trp, Leu, and His and containing 5 mM 3-AT, whereas the control group did not grow normally (Figure 9A). The yeast strain co-transformed with AmMYB111 and AmTTG1 also failed to grow normally. This indicates that AmMYB111 can directly interact with AmRGL2 in yeast, whereas AmMYB111 does not interact with AmTTG1.

#### 2.7.3. Luciferase Complementation (LCA)

To further confirm the interaction between the two in plants, LCA experiments were conducted in *Nicotiana benthamiana* leaves in this study. *AmMYB111* was cloned into the pCAMBIA1300-nLUC vector, and *AmRGL2* was cloned into the pCAMBIA1300-cLUC vector; both were co-transformed into tobacco leaves. The results showed (Figure 9B) that strong luciferase activity was detected in the regions co-expressing nLUC-*AmMYB111* and cLUC-*AmRGL2*, whereas only background-level fluorescence signals were detected in the control groups (nLUC + cLUC-*AmRGL2*, nLUC-*AmMYB111* + cLUC, and nLUC + cLUC). These experimental results clearly indicate that AmMYB111 and AmRGL2 undergo a specific protein–protein interaction within plant cells.

#### 2.7.4. Functional Analysis of AmRGL2

**Figure 10:**
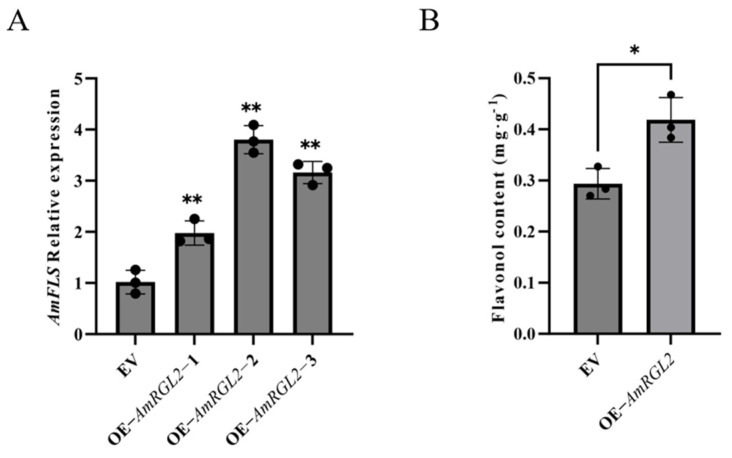
Functional exploration of *AmRGL2* genes: (**A**) relative expression level of *AmFLS* gene in OE-*AmRGL2* overexpressed lines; (**B**) flavonol content in OE-*AmRGL2* overexpressed lines. * Indicates significant difference (*p* < 0.05), ** indicates extremely significant difference (*p* < 0.01).

The experimental results showed that, in the experimental lines overexpressing *AmRGL2*, both *AmFLS* gene expression and flavonol content were significantly higher than those in the control group (EV) (Figure 10A,B); however, there was no significant effect on plant phenotype (Figure S8). qRT-PCR analysis confirmed that AmRGL2 transcript levels were significantly elevated in the OE-AmRGL2 lines compared to the EV control (Supplementary Figure S8B). Experimental results indicate that AmRGL2, as an interacting protein of AmMYB111, can promote the biosynthesis of flavonols.

## 3. Discussion

To investigate the accumulation patterns of flavonols in different organs of JHK, this study measured flavonol content in various vegetative organs and floral organs at different stages of flowering. The results revealed that flavonols are primarily concentrated in the floral organs of JHK and exhibit dynamic changes throughout the flowering period: levels peaked during stage T1, declined during stage T2, and rose again during stage T3. Among the vegetative organs, the content in stems and leaves was similar, while the content in roots was the lowest. This is consistent with the conclusions drawn by [1]. Regarding the distribution of flavonoids in JHK based on metabolomics analysis. The *AmFLS* and *AmF3′H* genes in JHK both positively regulate flavonol biosynthesis, with *AmFLS* being the key rate-limiting enzyme gene in the flavonol biosynthetic pathway. This is consistent with the view proposed by Frank et al. that upstream genes in the phenylpropanoid pathway are predominantly constitutively expressed, while the specific accumulation of flavonols is determined by the temporal regulation of downstream branch enzyme genes [32]. *FLS* and *F3′H* are key catalytic enzymes in the biosynthesis of plant flavonols, and their functions have been validated in plants such as Arabidopsis, grapes, and apples [33,34,35]. *F3′H* is responsible for the 3′-hydroxylation of dihydroflavonols, which determines structural diversity, while *FLS* catalyzes the conversion of dihydroflavonols to flavonols, serving as the key terminal enzyme in the synthesis [36,37]. It should be noted that the flavonol content in this study was measured as total flavonols using a colorimetric method (Section 4.6). The current study did not distinguish the specific flavonol species (e.g., kaempferol, quercetin, myricetin, and their glycosylated derivatives) that may be preferentially regulated by AmMYB111 and AmRGL2. Future metabolomic analyses using LC-MS will be required to resolve the precise compositional changes underlying the observed flavonol accumulation and to fully elucidate the regulatory specificity of these transcriptional regulators and their interacting partners.

In the plant flavonoid biosynthesis pathway, *MYB* transcription factors can act as transcriptional activators to positively promote flavonoid synthesis or as transcriptional repressors to negatively block the biosynthesis pathway, thereby maintaining a dynamic balance of flavonoid levels within the plant [38]. In Populus tomentosa, *MYB6* promotes the accumulation of anthocyanins and proanthocyanidins but also inhibits the formation of secondary cell walls [39], whereas *GhMYB6* in upland cotton enhances the plant’s resistance to Fusarium wilt by activating disease-resistant genes, demonstrating the differential differentiation of *MYB* transcription factors. In JHK, *AmMYB111* is a nuclear-localized R2R3-MYB transcriptional repressor whose expression level gradually increases during flower development, in stark contrast to the expression pattern of *AmFLS*. It directly binds to the *AmFLS* promoter and inhibits its transcription, thereby reducing *FLS* enzyme activity and negatively regulating flavonoid biosynthesis in JHK [40,41].

DELLA proteins generally lack DNA-binding ability and primarily regulate the activity of MYB transcription factors through protein–protein interactions [42,43]. In apples, the DELLA protein MdRGL2a directly interacts with the repressive MYB transcription factor MdMYB22. This interaction lifts the inhibitory effect of MdMYB22 on key downstream genes involved in anthocyanin biosynthesis, thereby significantly promoting anthocyanin accumulation in apple fruits [44]. In Arabidopsis, the core DELLA family protein RGL2 directly binds to MYBL2. By isolating MYBL2’s inhibitory effect on the MBW ternary complex, it activates the expression of genes related to flavonoid biosynthesis, helping plants establish a stable balance between GA growth signals and abiotic stress signals [31]. AmRGL2 belongs to the DELLA protein family and forms a specific protein–protein interaction with AmMYB111. AmRGL2 can upregulate the expression of the *AmFLS* gene and promote the accumulation of flavonols in JHK. Although our data demonstrate that AmRGL2 physically interacts with AmMYB111 and positively regulates flavonol accumulation, the precise molecular mechanism by which AmRGL2 modulates AmMYB111-mediated transcriptional repression remains to be elucidated. We cannot rule out the possibility that AmRGL2 acts through an indirect pathway or requires additional unknown factors. Future chromatin immunoprecipitation (ChIP) and electrophoretic mobility shift assays (EMSA) in the presence of AmRGL2 are needed to test whether AmRGL2 directly interferes with AmMYB111 binding to the AmFLS promoter.

## 4. Materials and Methods

### 4.1. Plant Materials and Strains

All experiments were conducted with at least three independent biological replicates, with each biological replicate consisting of three technical replicates. For VIGS and transient overexpression experiments, each treatment group comprised 20 individual seedlings, and the entire experiment was independently repeated three times. JHK is propagated from seed. In accordance with the experimental protocol, it was grown in a glass greenhouse. Samples were flash-frozen in liquid nitrogen and stored in a −80 °C freezer, with no fewer than three biological replicates retained for each experiment. As a dicotyledonous plant, JHK exhibits distinct morphological differences between cotyledons and true leaves during the seedling stage. For *Agrobacterium*-mediated silencing and transient overexpression experiments, cotyledons from seedlings at the dicotyledonous stage (approximately 7 days old) were selected as the injection material, as this stage features a well-developed system, a thinner epidermis, and higher transfection efficiency.

*Escherichia coli* DH5α-competent cells, *Agrobacterium* competent cells EHA105, and GV3101 were purchased from Beijing Qingke Biotechnology Co., Ltd.(Beijing, China). Yeast strains Y1H Gold and AH109 were purchased from Shanghai Weidi Biotechnology Co., Ltd. The vectors used, including TRV2, pCAMBIA2300, pAbAi, pGADT7, pGBKT7, pGreenII 62-SK, 0800-LUC, pCAMBIA1300-nLUC, and pCAMBIA1300-cLUC, were provided by the research group’s laboratory.

### 4.2. RNA Extraction, cDNA Synthesis, and PCR Amplification

Approximately 1 g of young plant leaf samples was weighed, placed in a grinding tube, and ground into a powder using a grinder (60 Hz, 30 s)(Shanghai Jingxin, Shanghai, China) while submerged in liquid nitrogen. RNA was extracted using the Polysaccharide-Polyphenol Plant Total RNA Extraction Kit (Vazyme, RC401-01). The following steps included tissue lysis, RNA adsorption, impurity removal, and elution. RNA was obtained from the leaves of JHK, and its quality and concentration were determined using a NanoDrop 2000 ultra-micro spectrophotometer. The extracted RNA can be used directly for reverse transcription or stored at −80 °C for future use.

Reverse transcription was performed using the cDNA Reverse Transcription Kit (Vazyme, R333) with the following reaction mixture: 4 µL of 5 × All-in-one qRT SuperMix, 1 µL of enzyme mixture, 1 µg of template RNA, and RNase-free deionized water added to a total volume of 20 µL. The mixture was thoroughly mixed. The protocol is as follows: 15 min at 50 °C, followed by 5 s at 85 °C.

Gene sequences were obtained from the research group’s transcriptomic data. ORF sequences were predicted using NCBI, and primers were designed using SnapGene software version 8.2 in accordance with primer design principles. The reaction mixture consisted of 25 µL ddH_2_O, 25 µL 2 × Phanta Flash Master Mix, 2 µL each of forward and reverse primers, and 1 µL cDNA. In the PCR instrument, after a 30 s pre-denaturation at 98 °C, 35 cycles were performed with 10 s of denaturation at 98 °C, 5 s of annealing at 58 °C, and 8 s of extension at 72 °C, followed by a final 1 min extension at 72 °C to terminate the reaction. Perform agarose gel electrophoresis at 140 V for 15 min. The products were recovered from the agarose gel using an agarose gel recovery kit (Tiangen, DP209).

The transcriptome sequencing was performed on the Illumina HiSeq 2000 platform (paired-end reads). A total of 97.64 Gb of clean data was obtained from 15 libraries (flowers, leaves, stems, roots, and seeds, with three biological replicates each). The clean data of each sample reached 5.81 Gb, with GC content ranging from 42.98% to 44.84% and Q30 bases > 92.73%. After assembly using Trinity, a total of 52,990 unigenes were obtained. Gene functions were annotated against multiple public databases, including NR, Swiss-Prot, KEGG, COG, KOG, GO, and Pfam. Differentially expressed genes were identified using the DESeq package version 1.8.3 with |log2(fold change)| ≥ 1 and *p* < 0.05 [1].

### 4.3. Plasmid Isolation and Vector Construction

For TA vector ligation, pCE2TA/Blumt-Zero was used. An amount of 4 µL of the PCR products was added to 1 µL of the TA vector, gently pipetted to mix, and incubated at 25 °C for 5 min. The ligation product was added to *E. coli* DH5α competent cells, incubated on ice for 30 min, heat-shocked at 42 °C for 90 s, cooled on ice for 2 min, and then added to 600 µL of antibiotic-free LB medium. The mixture was incubated in a shaking incubator at 37 °C and 200 rpm for 1 h. After harvesting and resuspending the cells, they were spread on Kana-resistant LB medium and incubated in an inverted incubator at 37 °C for 12 h. Single-colony colonies that tested positive by PCR were selected for sequencing and alignment, and the plasmid was extracted using the Plasmid Mini Prep Kit (TianGen, B0630C).

Use the primer design website (https://crm.vazyme.com/cetool/singlefragment.html, accessed on 15 August 2025.), select “Monoclonal Double Restriction Digestion,” enter the vector and gene sequences, select appropriate restriction sites, and design the homologous arms. The reaction mixture consists of the following: 20 µL deionized water, 25 µL 2 × Phanta Flash Master Mix, 2 µL each of upstream primer F and downstream primer R, and 1 µL AmFLS plasmid. Perform the PCR reaction. Upon completion, conduct gel electrophoresis and gel recovery experiments. The linearization reaction system for the empty vector is as follows: 1 μL each of two restriction endonucleases, 1000 ng of the empty vector plasmid, and ddH_2_O to a total volume of 20 μL. Centrifuge briefly to collect the solution at the bottom of the tube, then run the reaction in a PCR machine at 37 °C for 15 min and 80 °C for 20 min. After the reaction is complete, place the mixture on ice and set aside. The homologous recombination ligation reaction mixture consists of the following: 2 μL of linearized vector, 3 μL of gel-purified product, and 5 μL of CEMixV3 enzyme. Incubate in a PCR instrument at 50 °C for 15 min. After the reaction is complete, place the mixture on ice until ready for use.

### 4.4. Gene Silencing and Transient Overexpression

The TRV2 recombinant plasmid (5 μL) was added to Agrobacterium EHA10 competent cells. The mixture was cooled on ice for 5 min and then in liquid nitrogen for 5 min. This was followed by incubation in a metal bath at 37 °C for 2 min and then on ice for 2 min. Next, 900 μL of antibiotic-free LB medium was added, and the cells were incubated at 28 °C and 200 rpm for 3 h. The cells were then collected, resuspended, and spread onto LB medium containing kanamycin and rifamycin. After inverted incubation at 28 °C for 3 days, a single PCR-positive colony was selected for expansion. The *Agrobacterium* was placed in 200 mL of LB liquid medium containing kanamycin and rifamycin, and incubated overnight at 28 °C on a 200 rpm shaking incubator. It was centrifuged at 5500 rpm for 7 min to collect the bacterial cells.

The following stock solutions were prepared: (1) 1 M MgCl_2_: dissolve 20.321 g in 100 mL distilled water, autoclave, aliquot, and store at −20 °C; (2) 1 M MES: dissolve 21.325 g in 100 mL distilled water, filter-sterilize, aliquot, and store at −20 °C; (3) 50 mM AS: dissolve 0.981 g in 100 mL DMSO, sterilize by filtration, aliquot, and store at −20 °C. Resuspend the cells in a suspension (10 mM MgCl_2_, 10 mM MES, 200 µM AS, pH adjusted to 5.8) to an OD_600_ of 0.8–1.0. Mix the resuspended TRV1 with TRV2 and recombinant TRV2 in a 1:1 ratio, then let the mixture stand in the dark at 28 °C for 3–5 h. Using a disposable 1 mL syringe needle, gently prick the underside of the JHK cotyledon, avoiding the leaf veins. Remove the needle, draw up the settled bacterial suspension, and inject it along the wound into the leaf until the suspension spreads throughout the entire leaf. Wipe off any residual bacterial solution from the leaf. Incubate in the dark at 25 °C for 24 h, then maintain a normal photoperiod. After 15 days, observe phenotypic traits, record data, and collect samples.

In the gene overexpression experiment, *Agrobacterium* transformation and resuspension-mediated infection of the pCAMBIA2300 recombinant plasmid were performed as described above. The pCAMBIA2300 empty vector served as the control group. Phenotypic observations, data recording, and sample collection were conducted 3 days after leaf-back injection.

### 4.5. Subcellular Localization of the AmMYB111 Transcription Factor and a Dual-Luciferase Reporter System

The constructed pCAMBIA2300-*AmMYB111* recombinant vector and the pCAMBIA2300 empty vector were transformed into *Agrobacterium* GV3101 competent cells. Four to six healthy tobacco seedlings with well-developed leaves were selected. The bacterial suspension was drawn up into a 1 mL syringe and injected onto the underside of the tobacco leaves. The leaves were then cultured in the dark for 1 day, followed by 2 days of cultivation under light. Samples were then prepared into sections and examined under a confocal microscope to observe the GFP fluorescence signal and determine the subcellular localization of *AmMYB111*.

Using homologous recombination, the CDS of *AmMYB111* was cloned into the pGreenII 62-SK vector to serve as the effector, while the downstream *AmFL*S promoter was cloned into the pGreenII 0800-LUC vector to serve as the reporter. After obtaining the effector and reporter genes, they were transformed into *Agrobacterium* GV3101 (pSoup) to obtain positive clones. The control group consisted of empty pGreenII 62-SK and empty pGreenII 0800-LUC vectors. Following infection of tobacco plants, images were captured using an LB983 live-cell imaging system, and circular discs were excised from the tobacco leaves using a punch. Following the protocol provided by the dual luciferase reporter gene assay kit (Biyuntian, RG207). The LUC/REN ratio was measured and calculated using a microplate reader.

### 4.6. Determination of Gene Expression Levels and Flavonol Content

Avoid conserved gene regions and use GenScript (https://www.genscript.com) to design quantitative primers. Select the best design based on primer design principles as the quantitative primer, and verify it by detecting a single band via PCR gel electrophoresis. The internal control gene was *AmActin*. The expression levels of the target gene were detected using a real-time quantitative PCR system (Bio-Rad CFX96™ Real-Time System) and TB Green^®^ Premix Ex Taq™ II (TAKARA, RR820A). Real-time quantitative PCR reaction mixture: 5 μL TB Green, 1 μL cDNA template, 0.4 μL forward and reverse primers, 3.6 μL ddH_2_O. Amplification program: 95 °C for 30 s, 95 °C for 5 s, 55 °C for 15 s, 72 °C for 30 s, 95 °C for 5 s; repeat for 39 cycles.

The determination of flavonoid content was performed using the Adsbio Flavonoid Content Assay Kit (ADS-067-W). This method exploits the fact that plant flavonols form chelates with aluminum salts in neutral or slightly alkaline conditions in the presence of sodium nitrite, resulting in a maximum absorbance at 510 nm. The flavonoid content was then calculated using a colorimetric method.

The flavonoid content was calculated using a regression equation determined under standard conditions:

y = 1.5272x − 0.01 (R^2^ = 0.9994)

where x represents the concentration (mg/mL), and y represents the difference in absorbance (ΔA).

Flavonol content (mg/g fresh weight)= [(ΔA + 0.01) ÷ 1.5272 × V1] ÷ (W × V1 ÷ V2) = 0.65 × (ΔA + 0.01) ÷ W

V1: Volume of sample added to the reaction system, 0.1 mL; V2: Volume of extraction solution added, 1 mL; W: Sample mass, g.

### 4.7. Cloning of the AmFLS Gene Promoter

JHK DNA was extracted using the Plant Genomic DNA Extraction Kit (Tiangen, DP305) in accordance with the manufacturer’s instructions. Young JHK leaves were thoroughly ground into a powder using liquid nitrogen, followed by lysis, impurity removal, ethanol drying, and elution to obtain JHK DNA. The quality and concentration of the DNA were then determined using a NanoDrop 2000 ultra-micro spectrophotometer.

The *AmFLS* promoter was cloned using the chromosome walking method with the TaKaRa Genome Walking Kit (6108). Three specific primers were designed based on a validated known sequence region, oriented toward the unknown region to be amplified. SP2 was positioned inside SP1, and SP3 was positioned inside SP2. The distance between each pair of primers was set at 60–100 bp. The primer design principles were as follows: primer length of 22–26 bp, GC content of 45–55%, and Tm of 60–70 °C.

The first PCR reaction was performed using the AP Primer as the forward primer and the SP1 Primer as the reverse primer. The reaction mixture consisted of 1 μL DNA, 8 μL dNTP Mixture (2.5 mM each), 5 μL 10× LA PCR Buffer II (Mg^2+^ plus), 0.5 μL TaKaRa LA Taq (5 U·μL^−1^) 0.5 μL, AP Primer (100 pmol·μL^−1^) 1 μL, SP1 Primer (10 pmol·μL^−1^) 1 μL, and ddH_2_O 33.5 μL. The first PCR reaction protocol is shown in Table S1.

Dilute the product of the first PCR 200-fold, then use 1 μL of this diluted product as the template for the second PCR reaction. Use the AP Primer as the forward primer and the SP2 Primer as the reverse primer for the second PCR reaction. Except for the template and primers, the rest of the reaction conditions are the same as those for the first PCR reaction. The second PCR reaction protocol is shown in Table S2.

Dilute the 2nd PCR reaction mixture 300-fold, then use 1 μL of this mixture as the template for the 3rd PCR reaction. Use the AP Primer as the forward primer and the SP3 Primer as the reverse primer for the 3rd PCR reaction. In the reaction mixture, replace the template with the 2nd PCR reaction mixture diluted 300-fold; all other components remain the same as in the 1st PCR reaction. The 3rd PCR reaction protocol is identical to that of the 2nd PCR reaction. Isolate the PCR products using gel extraction; the subsequent ligation, transformation, and sequencing procedures are the same as described above. Design an upstream primer at a location distant from the ATG start codon in the obtained promoter sequence, and use PS3 as the downstream primer. Perform PCR cloning and sequencing using the DNA as a template. Align the sequence obtained after sequencing with the 5′ end sequence of the *AmFLS* gene; a match indicates that the promoter has been successfully cloned.

### 4.8. Yeast Monocultures (Y1H)

The truncated pro*AmFLS* construct was cloned into the pBait-AbAi vector, and the *AmMYB111* construct was cloned into the pGADT7 vector. An amount of 2 μg of the pBait-AbAi recombinant plasmid was linearized using the BstBI restriction enzyme. The digestion system is shown in Table S3, and the reaction conditions were 65 °C for 40 min.

Integrate 1 μg of linearized pBait-AbAi plasmid into the Y1H Gold strain by sequentially adding the following solutions: 240 μL of 50% polyethylene glycol (PEG), 36 μL of 1 M LiOAc, 10 μL of salmon sperm DNA (5 mg/mL) denatured at 100 °C, and 5 μL of the recombinant plasmid. Vortex immediately for 1 min; incubate on a 200 rpm shaker at 30 °C for 30 min; heat shock at 42 °C for 25 min, inverting the tube to mix during this process, and repeat 5 times; cool on ice for 5 min; centrifuge at 7000 rpm for 30 s, discard the supernatant, resuspend in 300 μL of sterile ddH_2_O, and spread 100 μL evenly onto SD/-Ura medium; incubate at 30 °C for 3–5 days. Yeast colonies were spotted onto SD/-Ura plates containing gradient concentrations of AbA (100, 150, 200, 400, 600, and 800 ng·mL^−1^) to determine the minimum inhibitory concentration (MIC).

Isolate single colonies from SD/-Ura plates, resuspend them in ddH_2_O, and spread 100 μL of the resuspended bacterial suspension onto SD/-Ura plates containing 100, 150, 200, 400, 600, and 800 ng/mL AbA. Isolate monoclonal cells with inhibited self-activation into 50 mL of YPDA medium, incubate at 30 °C on a shaking incubator at 200 rpm for 16–20 h, centrifuge at 4000 rpm at room temperature for 5 min to collect the cells, discard the supernatant, and resuspend the yeast in 60 mL of sterile deionized water. Add sequentially 240 μL of 50% PEG, 36 μL of 1 M LiAc, 10 μL of pre-denatured Carrier DNA, and 10 μL of pre-chilled pGADT7-*AmMYB111* plasmid, then vortex for 1 min. Incubate at 30 °C on a 200-rpm shaker for 30 min, heat shock in a 42 °C water bath for 25 min, invert and mix every 5 min, cool on ice for 5 min, and centrifuge at 5500 rpm for 5 min to collect the cells. Resuspend the cells in 300 μL of dd H_2_O, pipette 100 μL, and spread evenly onto SD solid medium without Leu and Ura. Incubate at 30 °C for 3–5 days. Additionally, transform pGADT7 into the same single-cell yeast using the same method as a control. Then, pick the monoclonal cells, resuspend and mix them with 0.9% NaCl, spot them onto 400 ng·mL^−1^ AbA + SD/−Leu−Ura screening medium, incubate at 30 °C for 2–5 days for detection, and photograph.

### 4.9. Yeast Double Mutant (Y2H)

The pGBKT7-AmMYB111 construct was first tested for auto-activation on SD/-Trp/-His medium and showed no detectable self-activation. Nevertheless, 5 mM 3-AT was included in the screening medium to ensure the stringency of the interaction assays (as shown in Figure 9A). The constructed recombinant plasmids pGADT7-*AmMYB111* and pGBKT7-*AmRGL2* were cotransformed into the AH109 yeast strain using the same method described in 1.2.4. Take the AH109 yeast strain and sequentially add 240 μL of 50% PEG, 36 μL of 1 M LiOAc, 10 μL of pre-denatured carrier DNA, and 10 μL each of the recombinant plasmids pGADT7-*AmMYB111* and pGBKT7-*AmRGL2*. Vortex immediately for 1 min, incubate on a shaking incubator at 30 °C and 200 rpm for 30 min, heat shock at 42 °C for 35 min (inverting the tube to mix 5 times during the heat shock), cool on ice for 5 min, centrifuge at 7000 rpm for 30 s, discard the supernatant, resuspend in 300 μL of 0.9% NaCl, and spread 100 μL evenly onto SD/-Trp-Lue medium; incubate for 3–5 days. Additionally, pGADT7 + pGBKT7-*AmRGL2*, pGADT7-*AmMYB111* + pGBKT7, and pGADT7 + pGBKT7 were cotrans- formed into the yeast strain AH109 as control groups.

Select 20 monoclonal clones from each combination, resuspend and mix them in 0.9% NaCl, and spot them onto plates containing 0, 5, 10, 25, 50, and 80 mM 3-AT + SD/-Leu-Trp -His screening medium. Incubate at 30 °C for 2–5 days, then perform the assay and take photographs.

### 4.10. Bimolecular Fluorescence Complementation and Assay of Flavonol Synthase (FLS) Activity

The constructed recombinant plasmids pCAMBIA1300-nLUC-*AmMYB111* and pCAMBIA1300-cLUC-*AmRGL2* were transformed into *Agrobacterium* GV3101 to form the experimental group. At the same time, pCAMBIA1300-nLUC + pCAMBIA1300-nLUC, pCAMBIA1300-nLUC-*AmMYB111* + pCAMBIA1300- cLUC, and pCAMBIA1300-nLUC + pCAMBIA1300-cLUC-*AmRGL2* as control groups. Subsequent shaking culture, resuspension, infection, and photography were performed as described above.

The activity of *FLS* in the samples was determined using mmbio’s Plant Flavonoid Synthase (*FLS*) Enzyme-Linked Immunosorbent Assay (ELISA) Kit (MM-098902).

### 4.11. Data Analysis

Raw data were processed using Excel 2021, figures were created using Photoshop 2019, and statistical analyses were performed using SPSS 23.0 or GraphPad Prism 10.0. All data are expressed as mean ± standard deviation (n = 3). For comparisons between two groups, Student’s *t*-test was applied. For comparisons among multiple groups, one-way analysis of variance (ANOVA) was performed, followed by Duncan’s multiple range test. Differences were considered significant at *p* < 0.05 (*) and highly significant at *p* < 0.01 (**). In this experiment, each treatment was replicated with 20 samples and performed in triplicate. For gene expression measurements, each data point represents a single measurement from a biological sample. For flavonoid content measurements, each data point represents the average of three technical replicates from a single biological sample.

## 5. Conclusions

In summary, this study systematically elucidated the tissue-specific accumulation patterns of flavonols in JHK and identified *AmFLS* as the key rate-limiting gene that positively regulates flavonol biosynthesis. We further demonstrated that *AmMYB111*, a nuclear-localized R2R3-MYB transcription factor, acts as a transcriptional repressor by directly binding to the *AmFLS* promoter and inhibiting its activity, thereby negatively regulating flavonol accumulation. Furthermore, we revealed a new layer of regulation involving the DELLA family protein AmRGL2, which specifically interacts with AmMYB111 and upregulates *AmFLS* expression, thereby enhancing flavonol biosynthesis. These findings reveal, for the first time, preliminary insights into the upstream transcriptional regulation of flavonol metabolism in JHK. This study not only deepens our understanding of the molecular mechanisms underlying flavonoid biosynthesis in Malvaceae plants but also provides potential targets for metabolic engineering and molecular breeding aimed at increasing the content of bioactive compounds in JHK and related medicinal crops.

## Data Availability

The transcriptome sequencing was performed on the Illumina HiSeq 2000 platform (paired-end reads). The datasets analyzed during the current study are available in the NCBI BioProject repository (PRJNA786451). The datasets used and/or analyzed during the current study are available from the corresponding author upon reasonable request due to privacy.

## Supplementary Materials

The following supporting information can be downloaded at: https://www.mdpi.com/article/10.3390/plants15152321/s1, Figure S1: Cloning of AmFLS and AmF3’H genes from JHK. Figure S2: Construction of virus-induced gene silencing vectors. Figure S3: Construction of transient overexpression vectors for target genes. Figure S4: Analysis of gene silencing and overexpression levels. Figure S5: Cloning and analysis of AmFLS gene promoter. Figure S6: Analysis of gene silencing and overexpression levels. Figure S7: Functional verification of *AmMYB111* transcription factor; Figure S8: Analysis of gene silencing and overexpression levels. Functional verification of AmMYB111 transcription factor. Table S1: Reaction program; Table S2: Reaction program; Table S3: The single digestion system; Table S4: Primer sequences used in this study.
