# Supplementary material for: Analysis of the Function and Regulatory Mechanisms of the AmFLS Gene, Encoding the Flavonol Synthase in Abelmoschus manihot L
Source: Plants (Basel). 2026 Jul 28;15(15):2321. doi: 10.3390/plants15152321 (PMC13468212; doi:10.3390/plants15152321)
Supplement: Supplementary file 1 [file plants-15-02321-s001.zip › plants-4406966-supplementary.pdf]

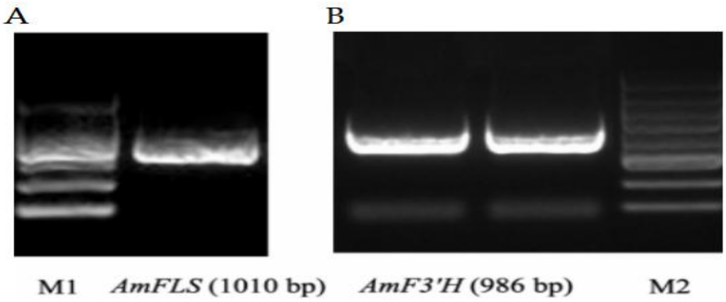

Figure S1. Cloning of *AmFLS* and *AmF3′H* genes from JHK. (**A**) *AmFLS* gene; (**B**) *AmF3′H* gene. M1: 2000bp DNA maker; M2: 5000 DNA maker.

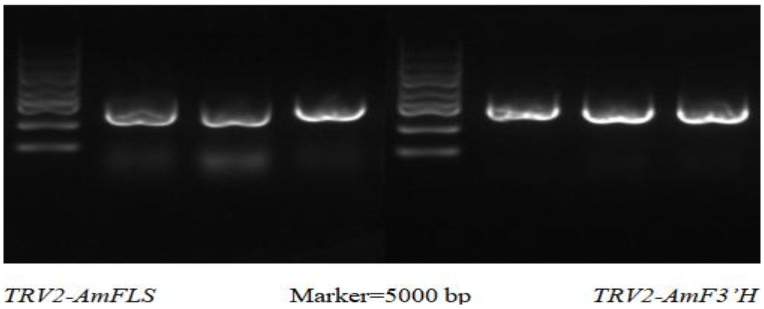

Figure S2. Construction of virus-induced gene silencing vectors.

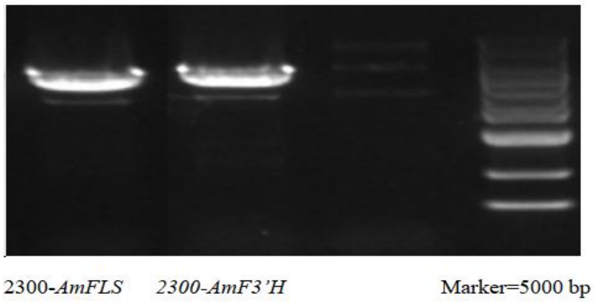

Figure S3. Construction of transient overexpression vectors for target genes.

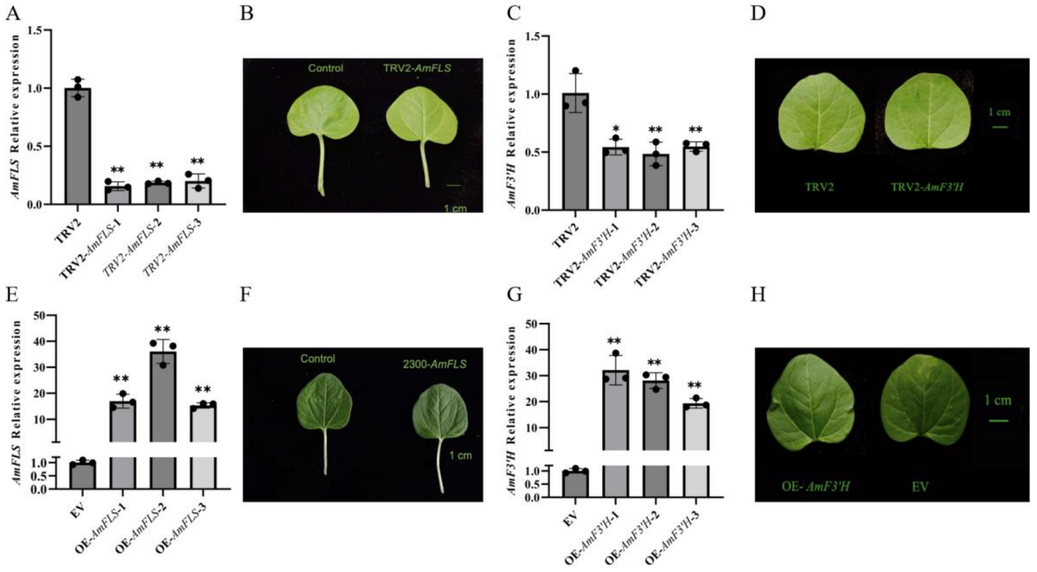

Figure S4. Analysis of gene silencing and overexpression levels. **(A)** TRV2-*AmFLS* gene expression levels; **(B)** TRV2-*AmFLS* plant phenotype map; **(C)** TRV2-*AmF3′H* gene expression levels; **(D)** TRV2-*AmF3′H* plant phenotype map; **(E)** OE-*AmFLS* gene expression levels; **(F)** OE-*AmFLS* plant phenotype map; **(G)** OE-*AmF3′H* gene expression levels; **(H)** OE-*AmF3′H* plant phenotype map.

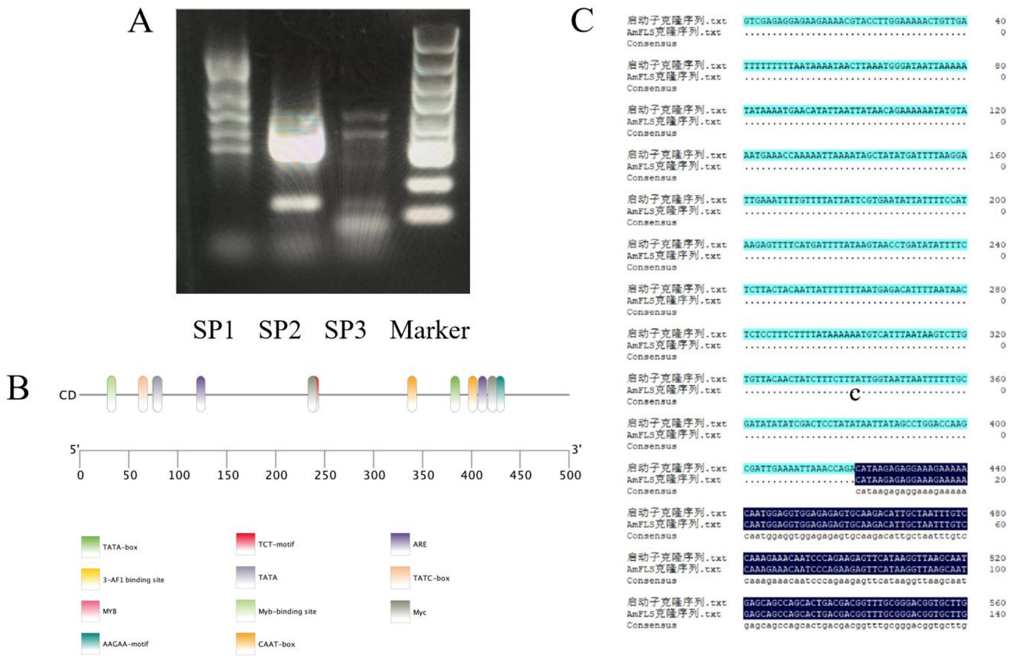

Figure S5. Cloning and analysis of *AmFLS* gene promoter. **(A)** Electrophoresis of PCR products from three rounds of genome walking for *AmFLS* gene promoter cloning. SP1, SP2, and SP3 represent the amplification products from the 1st, 2nd, and 3rd rounds, respectively, Marker is the DNA molecular weight marker, Marker = 5000 bp; **(B)** Distribution map of cis-acting elements in the *AmFLS* gene promoter; **(C)** *AmFLS* promoter sequence and its alignment with the *AmFLS* gene sequence.

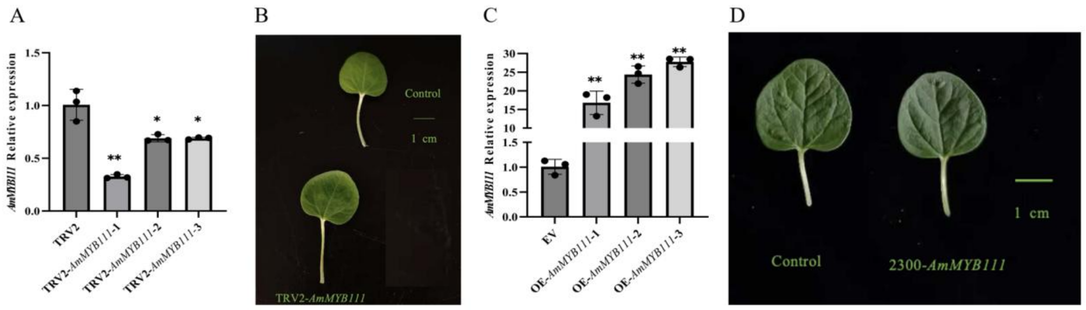

Figure S6. Analysis of gene silencing and overexpression levels. **(A)** TRV2-*AmMYB111* gene expression levels; **(B)** TRV2-*AmMYB111* plant phenotype map; **(C)** OE-*AmMYB111* gene expression levels; (D) OE-*AmMYB111* plant phenotype map;

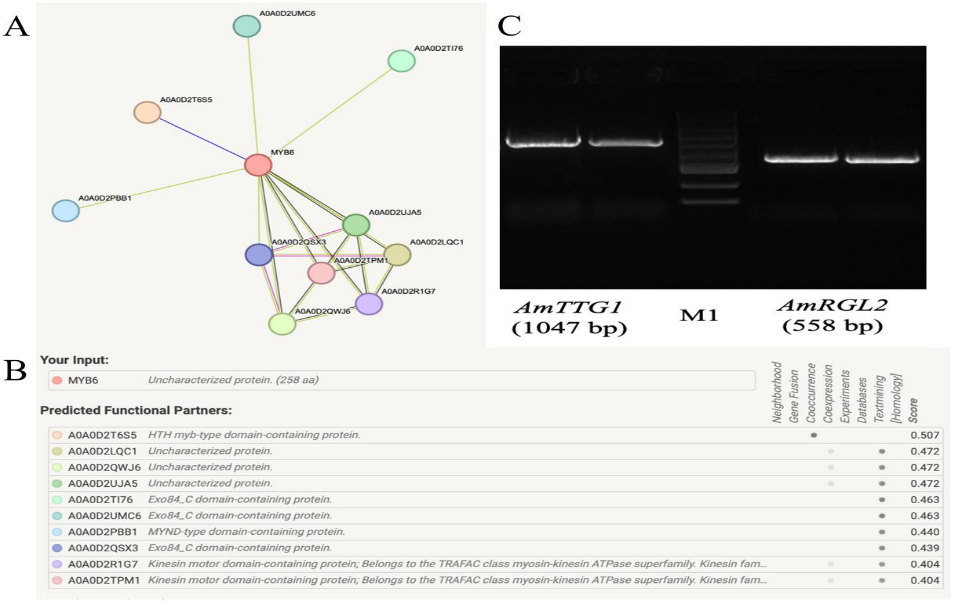

Figure S7. Functional verification of *AmMYB111* transcription factor. **(A)** Interaction network of *AmMYB111* homologous protein predicted by STRING database; **(B)** List of potential interacting proteins and their scores for *AmMYB111* homologous protein predicted by STRING database; **(C)** Electrophoretogram of *AmTTG1* and *AmRGL2* gene cloning, with M1 as 5000 bp DNA Marker.

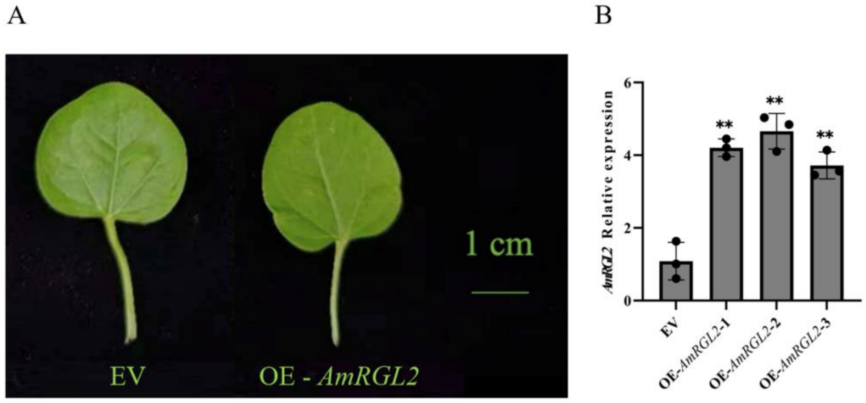

Figure S8. Analysis of gene silencing and overexpression levels. **(A)** OE-*AmRGL2* plant phenotype map; **(B)** OE-*AmRGL2* gene expression levels.

Table S1. Reaction program

Step	Temperature	Time	Cycles
1	94℃	1 min	
2	98℃	1 min	
3	94℃	30 s	
4	65℃	1 min	3～5：5 Cycles
5	72℃	2 min	
6	94℃	30 s	
7	25℃	3 min	
8	72℃	2 min	
9	94℃	30 s	
10	65℃	1 min	
11	72℃	2 min	
12	94℃	30 s	9～17：15 Cycles
13	65℃	1 min	
14	72℃	2 min	
15	94℃	30 s	
16	44℃	1 min	
17	72℃	2 min	9～17：15 Cycles
18	72℃	10 min	

Table S2. Reaction program

Step	Temperature	Time	Cycle
1	94℃	30 s	
2	65℃	1 min	
3	72℃	2 min	
4	94℃	30 s	
5	65℃	1 min	1～9：15 Cycles
6	72℃	2 min	
7	94℃	30 s	
8	44℃	1 min	
9	72℃	2 min	
10	72℃	10 min	

Table S3. The single digestion system

Reagent	Volume (μL)
pBait-AbAi	2 μg
10×BstBI buffer	4 μL
BstBI	1 μL
ddH_2O	Top up to 40 μL

Table S4. Primer sequences used in this study

Primer Name	Sequence（5'-3'）
pAbAi-F	GTTCCTTATATGTAGCTTTCGACAT
pAbAi-R	GTGTGTATTTGTGTTTGCGTGTC
pCAMBIA2300-F	CCATGTTGGCAAGCTGCTCT
pCAMBIA2300-R	CGTTGTAAAACGACGGCCAG

TRV2-F	GCTGTTTGAGGGAAAAGTAGAGAAC
TRV2-R	ACAAAAGACTTACCGATCAATCAAG
p-Green-SK-F	TGATGTGACATCTCCACTGACG
p-Green-SK-R	ATATGCTCAACACATGAGCGAA
0800-LUC-R	TCATAGCCTTATGCAGTTGCTCT
pGADT7-F	AATACGACTCACTATAGGGCG
pGBKT7-R	CATAAGAAATTCGCCCGGAATTAGC
pCAMBIA1300-nLUC-F	GAGCACGACACACTTGTCTAC
pCAMBIA1300-nLUC-R	CTCTTCATAGCCTTATGCAGTTG
pCAMBIA1300-cLUC-F	AAGAGATCGTGGATTACGTCG
pCAMBIA1300-cLUC-R	CAAGCACAACAAATGGTACAAG
sp-3	GCTTCCACAATGGCCTTCACCAA
sp-2	GCTTCCACAATGGCCTTCACCAA
sp-1	CTTCTTGCGGGAGCTCGAAGAAAG
pro*AmFLS*-F	CCTTGGAAAAACTGTTGATTTTTTTTTAAT
pro*AmFLS*-R	CTTGGTCCAGGCTATAATTATATAGGAG
Pabai-pro*AmFLS*-SmaI-F	AATTCGAGCTCGGTACCCGGGAAATCCCTTAACGTGAGTTTTCGT
Pabai-pro*AmFLS*-xhoⅠ-R	ATACAGAGCACATGCCTCGAGTCTGCGCTCTGCTGAAGCC
0800-pro*AmFLS*-SaⅠⅠ	GGGCCCCCCCTCGAGGTCGACACCTTGGAAAAACTGTTGATTTTTT
0800-pro*AmFLS*-BamHⅠ	CGCTCTAGAACTAGTGGATCCCACAAGACTTATTAAATGACATTTTTTATAAA
AmFLS-F	CATAAGAGAGGAAAGAAAAACAA
AmFLS-R	GATACGGCAGAGTTCAACAA
pCAMBIA2300-*AmFLS*-kpnⅠ	ATTTGGAGAGGACAGGGTACCATGGAGGTGGAGAGAGTGCAAG
pCAMBIA2300-*AmFLS*-xbaⅠ	GGTACTAGTGTCGACTCTAGAATTATGTTTGAGAAGGGTCTTGTTGA
TRV2-*AmFLS*-EcorⅠ	GTGAGTAAGGTTACCGAATTCTCCAAAATCTACTCTGACTATTGTGCC
TRV2-*AmFLS*-kpnⅠ	GAGACGCGTGAGCTCGGTACCGATACGGCAGAGTTCAACAACAAA
AmFLS-qRT-PCR-F	CAGATGTTTGTCGTTGGGCTTAG
AmFLS-qRT-PCR-R	GGCACGGCGGATAGTAGTTG
AmF3′H-F	ACGTAAACGTAAACGTCATGAA
AmF3′H-R	AATCATGGATTCCTTTGTTCTCTAT
pCAMBIA2300-*AmF3′H*-kpnⅠ	ATTTGGAGAGGACAGGGTACCATGGATTCCTTTGTTCTCTATTCCC
pCAMBIA2300-*AmF3′H*-xbaⅠ	GGTACTAGTGTCGACTCTAGAGTACGCCTGCTGCGATAACC
TRV2-*AmF3′H*-EcorⅠ	GTGAGTAAGGTTACCGAATTCACGTAAACGTAAACGTCATGAAGAA
TRV2-*AmF3′H*-kpnⅠ	GAGACGCGTGAGCTCGGTACCTTGTTGACGGCGACGTTAGTG
AmF3′H-qRT-PCR-R	TGTTGCCGGACAAACTGAAC
TRV2-*AmMYB111*-EcorⅠ	GTGAGTAAGGTTACCGAATTCCTCCTTGGTCACGATATGTCAATG
AmMYB5-F	GTGAAATTCCGTTTCCGTCG
AmMYB5-R	TGGAAGGCAATTATCCTAACCGT
pGreenII 62-SK-*AmMYB5*-Not1	GCCATGGAGGCCAGTGAATTCGTGAAATTCCGTTTCCGTCG
pGADT7-*AmMYB111*-EcorⅠ	GCCATGGAGGCCAGTGAATTCATCCGTCTCTCTCTGGCTTTCA
pGADT7-*AmMYB111*-BamHⅠ	CAGCTCGAGCTCGATGGATCCTTTTTTTTATGCCTCTGTTTTAAATTAGA
AmGL3-qRT-PCR-F	CCATCAGCAGCATTGTCTCC
AmGL3-qRT-PCR-R	AGCCAAATAGGCTGACCACT
AmTTG1-qRT-PCR-F	CGACACTTTCCTTCGACCAC

AmTTG1-qRT-PCR-R	CTCGAACTTCCCAGAGACGA
AmRGL2-qRT-PCR-F	TTGCAGCCATGTTCGATTCA
AmRGL2-qRT-PCR-R	ATGGCTCTCCATCCTGCTTT
AmMYB111-qRT-PCR-F	CGAAATCAAACTACTTAC
AmMYB111-qRT-PCR-R	AATACTATGTTTCTGCAGA
AmGL3-F	ATGGCTACTGAGTTCCAAAATC
AmGL3-R	TACACAAAGGTTAAAGATTGAACTCTT
AmTTG1-F	ATGGAGAATTCGACTCAAGAATCAAAC
AmTTG1-R	TCAAACTTTGAGAAGCTGCATTTTGT
AmRGL2-F	CCATTAAGAATGATATGAATGAGTCAG
AmRGL2-R	GAGTCAGTCCTATCAGATACATTG
AmMYB111-F	ATGCTGCTCATTTTTAAGGCTG
AmMYB111-R	AGATTCCAATCTGTATTATGATGAAA
pCAMBIA2300-*AmGL3*-kpn Ⅰ	ATTTGGAGAGGACAGGGTACCATGGCTACTGAGTTCCAAAATCAA
pCAMBIA2300-*AmGL3*-Xba Ⅰ	GGTACTAGTGTCGACTCTAGATTACACAAAGGTTAAAGATTGAACTCTTT
pCAMBIA2300-*AmTTG1*-kpn Ⅰ	ATTTGGAGAGGACAGGGTACCATGGAGAATTCGACTCAAGAATCAA
pCAMBIA2300-*AmTTG1*-xba Ⅰ	GGTACTAGTGTCGACTCTAGATCAAACTTTGAGAAGCTGCATTTT
pCAMBIA2300-*AmRGL2*-kpn Ⅰ	ATTTGGAGAGGACAGGGTACCATGGTGTTTCATTTGAACACTTTGG
pCAMBIA2300-*AmRGL2*-xba Ⅰ	GGTACTAGTGTCGACTCTAGATCAGATACATTGCCATGCAGATG
pCAMBIA2300-*AmMYB111*-pn Ⅰ	ATTTGGAGAGGACAGGGTACCATGCTGCTCATTTTTAAGGCTGT
pGADT7-*AmTTG1*-BamH Ⅰ	CAGCTCGAGCTCGATGGATCCTCAAACTTTGAGAAGCTGCATTTT
pCAMBIA1300-cLUC-*AmRGL*2-Asc Ⅰ	GTTAATTAATCGGACGGCGCGCCTCAGATACATTGCCATGCAGATG
